# Insights into the electronic, magnetic structure, and photocatalytic activity of Y_2_CuMnO_6_ double perovskite†

**DOI:** 10.1039/d4ra06357k

**Published:** 2025-01-30

**Authors:** Bhagyashree Munisha, Lokanath Patra, Jyotirmayee Nanda, Sneha Mondal

**Affiliations:** a Centre for Nanoscience and Nanotechnology, ITER, S‘O’A Deemed to be University Khandagiri Bhubaneswar 751030 Odisha India jyotirmayeenanda@soa.ac.in; b G. W. Woodruff School of Mechanical Engineering, Georgia Institute of Technology Atlanta GA 30332 USA; c Department of Physics, ITER, S‘O’A Deemed to be University Khandagiri Bhubaneswar 751030 Odisha India; d Department of Physics and Nanotechnology, Faculty of Engineering and Technology, SRM Institute of Science and Technology Kattankulathur Chennai Tamil Nadu 603203 India

## Abstract

This research aims to develop Y_2_CuMnO_6_ double perovskite, using a citrate auto combustion method, to be used as a photocatalyst for the degradation of organic dyes and antibiotics. XRD and Raman characterization revealed the synthesis of pure-phase Y_2_CuMnO_6_ double perovskite. The X-ray photoelectron spectroscopy results show the presence of +4 and +2 oxidation states of Mn and Cu ions. Our electronic structure analysis, Mott–Schottky, and UV-vis-NIR analysis suggest strong UV and visible region absorption. Our density functional theory analysis reveals that Y_2_CuMnO_6_ exhibits characteristics of a ferromagnetic semiconductor with low effective mass. The Jahn–Teller active Cu^2+^ ion induces local distortions, contributing to the stabilization of the low-symmetry monoclinic structure (*P*2_1_/*n*). The ferromagnetic superexchange mechanism is attributed to the overlap between the empty e_g_ band of Mn^4+^ and the partially filled e_g_ band orbital of Cu^2+^. The Y_2_CuMnO_6_ double perovskite resulted in degradation efficiencies of 99%, 96%, and 95% of rhodamine B, methylene orange dyes, and tetracycline antibiotics, respectively. This study reveals that the Y_2_CuMnO_6_ double perovskite achieved enhanced photocatalytic activity compared to commercial P25 TiO_2_. It demonstrated the remarkable photocatalytic properties of the Y_2_CuMnO_6_ catalyst indicating its significant potential for diverse environmental applications.

## Abstract 2

We develop Y_2_CuMnO_6_ double perovskite using a citrate auto combustion method, to be used as a photocatalyst for the degradation of organic dyes and antibiotics.

## Graphical abstract

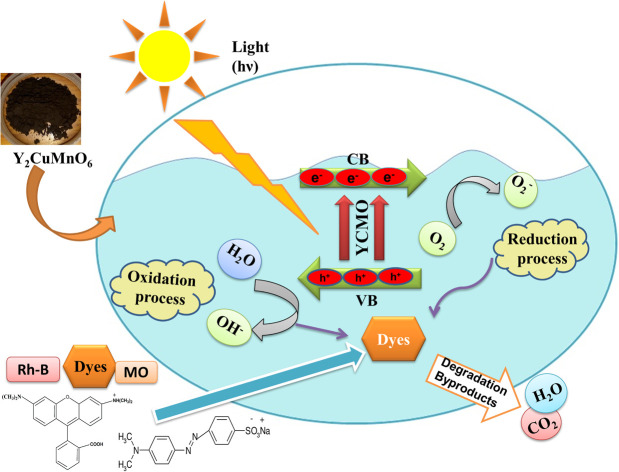

## 1. Introduction

The food and textile sectors are a major source of many types of dye that are discharged into the environment, which has led to concerns about dye pollution. Since dyes are very soluble, they readily move through water and can combine to generate compounds that are mutagenic, hazardous, and extremely poisonous to living things. The present focus of research attention is on determining the best mechanism for the breakdown of dye molecules.^1–3^ Solar light-driven photocatalysis, an effective method that uses light energy to start chemical reactions with an appropriate catalyst, has grown in popularity due to its application in sustainable energy conversion and environmental clean-up for the degradation of dye molecules.^4–8^

In the area of advanced materials research, double perovskites (A_2_BB′O_6_) have emerged as intriguing candidates for a wide range of technological applications, due to their unique electrical and structural characteristics.^9–12^ Rare-earth-based double perovskites have drawn a lot of attention due to their exceptional field-sensitive, magnetic, multiferroic, and strong magnetic–electrical coupling properties, with potential applications in a variety of fields, including magnetocaloric materials and commercial applications.^13,14^ Y_2_CuMnO_6_ (YCMO) stands out as an interesting member of the double perovskite family;^15,16^ the combination has exceptional promise as a photocatalyst, particularly for applications in harvesting solar energy and reducing environmental concerns. The ability of YCMO to absorb photons effectively throughout a wide spectral range, including visible light, makes it an appealing choice for generating photocatalytic processes. Furthermore, its distinct electronic band structure and crystal lattice arrangement lead to improved charge separation and mobility, which are critical features in generating better photocatalytic performance. Researchers are constantly investigating innovative synthesis approaches to improve the efficiency and stability of this material, and to overcome obstacles associated with photoexcited charge recombination, expanding its scope of use to varied photocatalytic processes.

Recently, there has been an upward trend in interest in double perovskite oxide structures for photocatalytic applications; however, there are not many publications on their use. Most studies on M_2_CuMnO_6_ compounds have been focused on La_2_CuMnO_6_,^17,18^ but the research on M_2_CuMnO_6_ (M = Y, Nd, Sm, Eu, Gd, and Pr) is rare.^19–21^ In 2020, Mansoorie *et al.* synthesized YCMO double perovskite manganite using a wet chemical sol–gel process to evaluate its electrochemical behaviour.^15^ Two years later Saha *et al.* synthesized YCMO double perovskite using a solid-state method to investigate the magnetic and dielectric properties of the material.^16^ Additionally, compared to other transition metals like Ni and Co, Cu-based materials have demonstrated superior photocatalytic performance, *i.e.*, higher recycling stability and reduced charge transfer resistance. In addition, the magnetic, dielectric, and electrochemical properties of Y-based double perovskites like Y_2_NiMnO_6_, Y_2_FeMnO_6_, and Y_2_CrMnO_6_, have been studied experimentally.^22–24^ The citrate auto-combustion synthesis method and photocatalytic behaviour of YCMO have not yet been reported.

Magnetic double perovskites exhibit a diverse array of significant properties, including high Curie temperatures, superconductivity, magnetocaloric effects, multiferroic behaviours, and magnetoresistance, stemming from their excellent magnetic characteristics.^25,26^ Recent investigations have revealed the potential of specific compositions like La_2_NiMnO_6_ ^27^ and La_2_CoMnO_6_ ^28^ as ferromagnetic semiconductors, positioning them as promising materials for spintronic devices. Subsequent studies have shown that substituting Co with Cu in La_2_CoMnO_6_ enhances its suitability for spintronic applications. The magnetic properties of Cu–Mn-based double perovskite oxides with 3+ cations such as La^3+^ and Y^3+^ in the A-site, are of particular interest due to Mn’s unusual oxidation state of +4 and the presence of Jahn–Teller active Cu^2+^ ions. Synthesis efforts targeting lanthanum copper manganate double perovskite La_2_CuMnO_6_, have revealed diverse behaviour, including ferromagnetism and magnetoresistance.^29,30^ Additionally, Mansoorie *et al.*^15^ reported that YCMO exhibits a monoclinic structure with *P*2_1_/*n* symmetry, attributing its ferromagnetism to superexchange coupling between the Cu^2+^ and Mn^4+^ ions. These compounds generally exhibit ferromagnetic and insulating behaviour, often forming ferromagnetic clusters, although antisite disorder can lead to antiferromagnetic ordering at lower temperatures, as exemplified by the orthorhombic antiferromagnetic structure observed in YCMO with randomly distributed Cu^2+^ and Mn^4+^ ions at the B site.^16^

The integration of experimental and theoretical approaches here in our paper on YCMO double perovskite, establishes a comprehensive understanding of their properties and potential applications. The crystalline structure, particle size, and surface morphology, all impact the properties of the material, which in turn impact the synthetic route. Consequently, several techniques, including the high-temperature solid-state method, the Pechini method, and the sol–gel citrate method, have been used to synthesize double perovskite materials. The current research focuses on the fabrication of the YCMO double perovskite material using the citrate auto-combustion method. The exploration of its structural, microstructural, optical, and electronic properties is characterized using XRD, RAMAN, FESEM, XPS, Mott–Schottky and UV-vis analysis. It has the potential to be utilized as a visible-light-driven photocatalyst.^15^ Density functional theory analysis was used to explore if YCMO exhibits characteristics of a ferromagnetic semiconductor with low effective mass. To the best of our knowledge, this work is the first to examine density functional theory analysis and photocatalytic activity of the YCMO double perovskite, when applied to the degradation of rhodamine B (Rh-B), methylene orange (MO) dyes and tetracycline (TC) antibiotics under visible light irradiation.

## 2. Methods

### 2.1 Experimental details

A simple auto-combustion method was used to synthesize YCMO double perovskite: 3 g of yttrium nitrate hexahydrate [Y(NO_3_)_3_·6H_2_O], 1.14 g cupric acetate monohydrate [Cu(CH_3_COO)_2_·H_2_O], and 1.14 g manganese acetate tetrahydrate [Mn(CH_3_COO)_2_·4H_2_O], were dissolved in deionized water, using 0.88 mL of ethylene glycol and 6.6 g of citric acid as surfactants. The citrate solution approach was chosen because of its capacity to improve grain structure uniformity and assure the purity of the target phase in all samples. The resultant solution was heated to 150 °C to create a fluffy mass, then subsequently dried and calcined for 12 hours at 1050 °C to obtain the desired YCMO double perovskite.

### 2.2 Computational details

Density functional theory (DFT) calculations were conducted using the Vienna *Ab initio* simulation package (VASP),^31^ employing projected-augmented wave (PAW)^32^ pseudopotentials, as described by the Perdew–Burke–Ernzerhof form of the generalized gradient approximation (PBE-GGA).^33^ The energy cut-off for the plane wave basis set was consistently set at 600 eV for all computations. Energy and force convergence criteria were established at 10^−5^ eV and 0.01 eV Å^−1^, respectively. The sampling of the Brillouin zone utilized the Monkhorst–Pack^34^ scheme with a grid of dimension (6 × 6 × 5). To account for the strongly localized d electrons of Cu and Mn, Hubbard *U* values of 5 eV were applied.^35,36^ For a precise determination of the band gap, the HSE06 hybrid functional^37^ was employed in our calculations.

### 2.3 Photocatalytic experiments

The photocatalytic activity test for the YCMO sample was done for the breakdown of Rh-B and MO dyes under direct sunlight, between 10 AM and 4 PM in June. For this experiment, 100 mL DI water was used to make dye solutions at 5, 10, and 20 ppm independently. Before starting the photocatalytic reaction, the dye solutions were sonicated for 10 minutes. After that, 50 mg of the photocatalyst was added to the solutions to achieve adsorption–desorption equilibrium, further sonication was performed for 45 minutes in a dark environment. The solutions had been stirred with a magnetic stirrer and exposed to sunlight once they had reached equilibrium. The degradation experiment was conducted without the use of any cut-off filter. We wanted to simulate real-world circumstances and evaluate the photocatalytic efficiency of YCMO double perovskite in conditions that are pertinent to the environment using natural sunlight. The detailed experimental setup has been discussed in our previous work.^38^

## 3. Results and discussion

### 3.1 Structural and microstructural studies

**Fig. 1:**
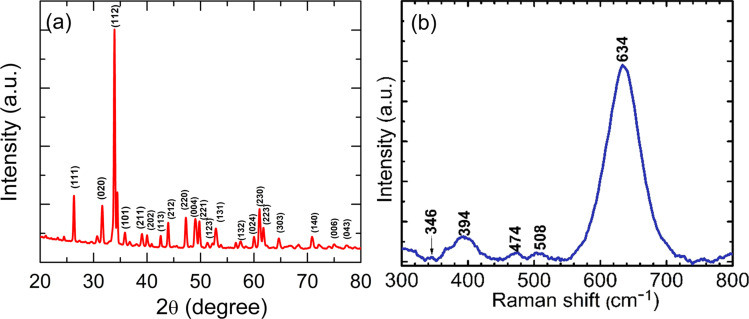
(a) XRD pattern and (b) Raman spectra of YCMO.

Powder-XRD (X-ray diffraction) analysis (Rigaku Ultima IV powder diffractometer) is used to determine the crystal structure and phase composition of materials. Fig. 1(a) shows the XRD pattern of the YCMO double perovskite, confirming the monoclinic crystalline structure. The XRD peak locations and intensities observed were consistent with those recently reported by Saha *et al.*^15,16^ The average crystalline size (*D*), microstrain (*ε*), and dislocation density (*δ*) of the prepared nanoparticles using Debye–Scherrer’s formula, were estimated as 33 nm, 0.00059, and 0.0149, respectively. Raman active (Model-Renishaw) modes located at 634, 394, and 346 cm^−1^ (see Fig. 1(b)) are ascribed to symmetry *A*_g_, and those at 508 and 474 cm^−1^ to symmetry *B*_g_, of the monoclinic structure of the YCMO double perovskite, thus confirming the absence of any secondary phases.

**Fig. 2:**
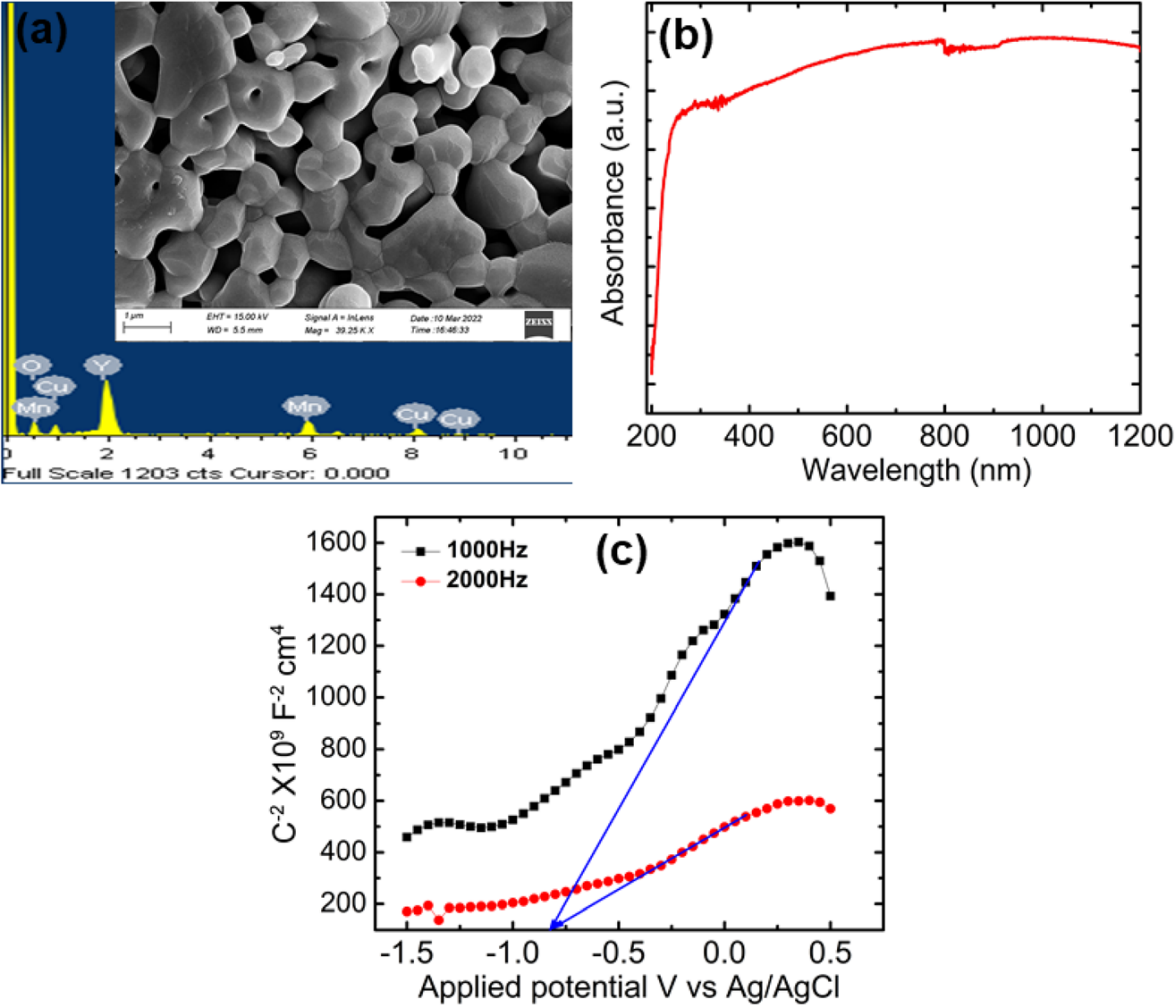
(a) Elemental analysis from EDX image (inset: FESEM image) of YCMO double perovskite, (b) UV-vis-NIR absorbance spectrum, and (c) Mott–Schottky plots of YCMO double perovskite.

FESEM (Zeiss Gemini 300) analysis was used to examine the size and surface morphology of YCMO double perovskite, as shown in the inset of Fig. 2(a). The high calcination temperature and strong reactivity of the nanoparticles caused the particles to aggregate, leading to comparatively large particle sizes and a highly porous material. The composition of the YCMO double perovskite was determined by EDX analysis. Fig. 2(a) validates the structure by displaying the existence of Y, Cu, Mn, and O peaks in the sample.

The electron probe microanalysis (EPMA) (model-EPMA-1720 HT) and EDX data of the YCMO sample provide insights into its elemental composition and spatial distribution. The qualitative analysis confirms the presence of oxygen (O), manganese (Mn), copper (Cu), and yttrium (Y), essential elements for this double perovskite structure. The EPMA analysis employed the ZAF correction method, with a gold (Au) coating applied to enhance conductivity. A summarized comparison is presented in Table 1. The EPMA and EDX data were compared with the theoretical nominal values of 45.32%, 16.2%, 14.01%, and 24.47% for Y, Cu, Mn, and O, respectively. While the EPMA and EDX data demonstrate slight deviations for Y, Cu, and O elements, these are within acceptable ranges, reflecting variations arising from measurement techniques and sample preparation. However, the discrepancies in the wt% of the Mn element may be further investigated using the more precise measurement technique, (Inductively Coupled Plasma) ICP spectroscopy. The combined analysis confirms the near-stoichiometric composition of YCMO, validating the synthesis process and its potential for functional applications.

**Table 1:** Comparison of elemental composition: nominal, EDX, and EPMA data analysis

Element	Nominal conc. (wt%)	EDX analysis (wt%)	EPMA analysis (wt%)
Y	45.32	44.05	31.51
Cu	16.20	13.03	17.27
Mn	14.01	27.45	31.09
O	24.47	15.47	20.04

### 3.2 Optical analysis

It is well known that to achieve maximum photoactivity, a photocatalyst must be able to absorb light. When a photon with an energy higher than the semiconductor band gap energy is absorbed, the photocatalysis process can begin in a semiconductor. The interband transition that results in the creation of holes in the valence band (VB) and electrons in the conduction band (CB) becomes feasible by this photoexcitation phenomenon. Therefore, we have examined the room temperature UV-vis-NIR spectrum (Model-UV-3101PC, Shimadzu Corporation, Japan) analysis of the synthesized YCMO to explore its light absorption characteristics and band gap energy. As displayed in Fig. 2(b), the absorbance spectrum exhibits a broad absorption starting from the wavelength ranging from the mid-UV to visible and near-infrared regions. This is expected as the YCMO powder is black in colour, supported by the similar broad spectrum observed in the case of black-TiO_2_ powder.^39^ This broad absorption range in the visible wavelength region shows that the YCMO double perovskite may effectively absorb solar energy, resulting in the production of photoinduced electron–hole pairs. Furthermore, a small absorption drop (like a step) appearing at the wavelength of 820 nm may be attributed to oxygen vacancies with trapped electrons.^39^ As a result of these features, the YCMO double perovskite may be considered a potential candidate for different photovoltaic and photocatalytic applications.^40^

The Mott–Schottky plots (measured at 1 kHz and 2 kHz in the dark) were used to identify the band-edge positions and semiconductor types (either n-type or p-type) based on the slope sign of the inverse square of the space charge capacitance (*C*^2−^) *versus* the applied bias (*V*) at the sample/electrolyte interface. As shown in Fig. 2(c), YCMO is identified as an n-type semiconductor. By extrapolating the linear portion of these curves to the potential axis, the flat band potentials were determined to be −0.82 V for YCMO (*vs.* Ag/AgCl, pH = 13.6). When converted to the standard hydrogen electrode (NHE) scale at pH = 0, these values correspond to −1.014 V for the CB value of the YCMO sample. The flat-band potentials can be roughly interpreted as conduction band edges.

### 3.3 XPS analysis

**Fig. 3:**
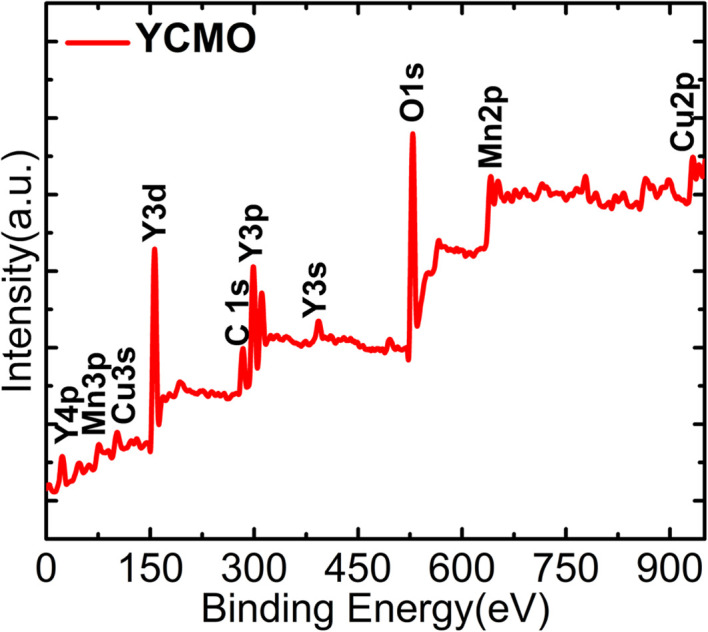
XPS survey scan spectrum of YCMO double perovskite.

**Fig. 4:**
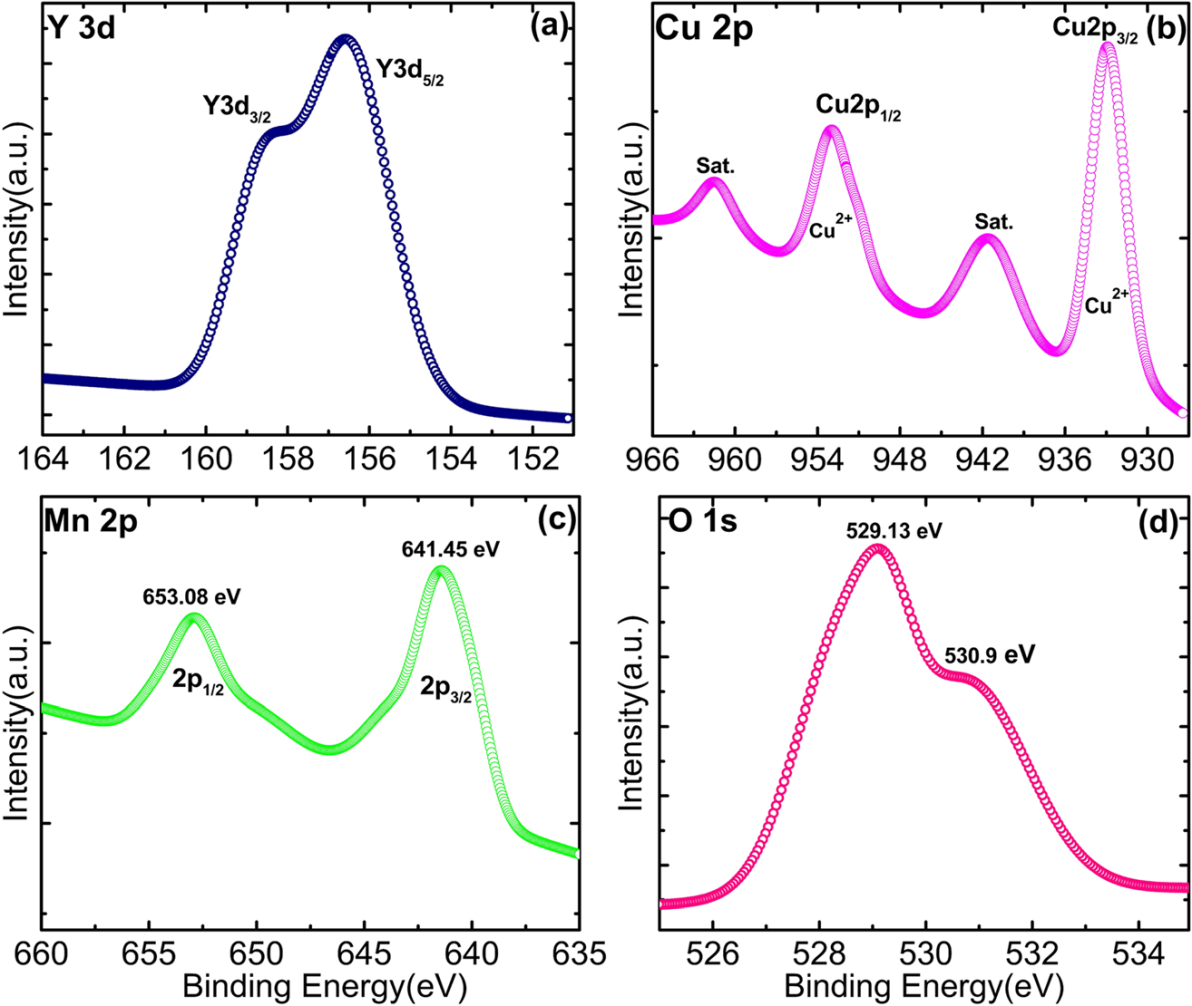
XPS spectra of (a) Y 3d, (b) Cu 2p, (c) Mn 2p, and (d) O 1s, in the YCMO double perovskite.

The elemental valence state and chemical composition of the YCMO sample were estimated using X-ray photoelectron spectroscopy (XPS) (model- PHI 5000 VersaProbe III). XPSpeak41 software was used to fit all of the collected spectra with linear background subtraction. The survey scan spectrum of the YCMO double perovskite is shown in Fig. 3, where the peaks represent Y, Cu, Mn, and O elements. There are no other impurity peaks in the survey scan spectra except the C 1s peak at 284.9 eV used to calibrate binding energies. So, the appearance of elements like Y, Cu, Mn, and O found in the synthesized material is corroborated by EDX results and XPS spectra. The two principal yttrium characteristic peaks, centred at 156.6 and 158.4 eV (shown in Fig. 4(a)), may be ascribed to Y 3d_5/2_ and Y 3d_3/2_, respectively, and their band splitting, *Δ* = 1.8 eV was found to be consistent with the value indicated in the literature.^41^ The presence of two peaks confirms the Y^3+^ oxidation state in the YCMO sample. Fig. 4(b) displays the XPS spectra for Cu 2p using the linear backdrop. For Cu 2p_1/2_ and Cu 2p_3/2_, the spectrum exhibits satellite peaks in addition to a double peak structure. Cu 2p_3/2_ is represented by the peak with a binding energy of 933.05 eV, while the Cu 2p_1/2_ level with a spin–orbit coupling of 20 eV is represented by the peak at 953.01 eV. In addition to the Cu 2p_3/2_ and Cu 2p_1/2_ peaks, the spectrum displays satellite peaks indicating the existence of Cu^2+^ ions at 941.5 eV and 961.48 eV, respectively. The two principal Mn characteristic peaks, centred at 641.5 and 653.08 eV (shown in Fig. 4(c)), may be ascribed to Mn 2p_3/2_ and Mn 2p_1/2_, respectively. The presence of two peaks confirms the Mn^4+^ oxidation state in the YCMO sample. Fig. 4(d) depicts the O 1s spectrum with two peaks at 529.13 and 530.9 eV that might be assigned to surface-adsorbed oxygen and oxygen–metal bonds, respectively.

### 3.4 Density functional theory studies

#### 3.4.1 Structural properties

**Fig. 5:**
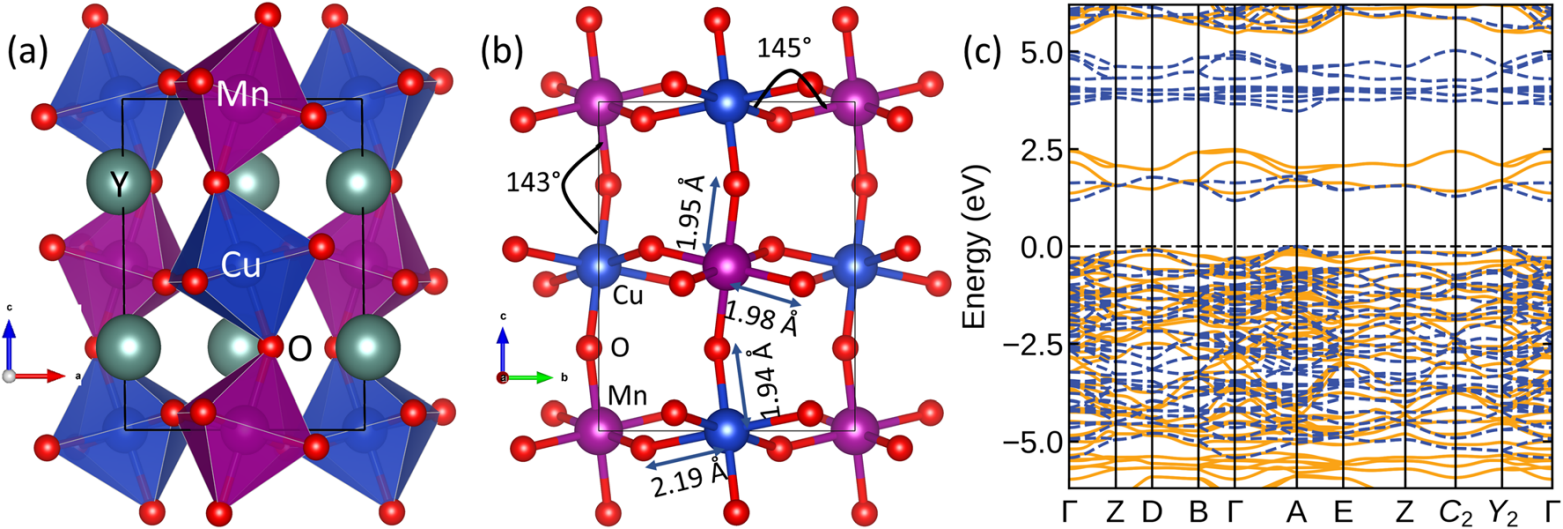
(a) The optimized crystal structure and (b) distortion of CuO_6_ and MnO_6_ octahedra in YCMO; the Cu/Mn–O bond lengths and Cu–O–Mn bond angles are given. The significant difference between in-plane and out-of-plane Cu–O bonds indicates compression of the CuO_6_ octahedron. (c) The spin-polarized band structure in the FM state with PBE-DFT level of theory. The yellow (solid) and blue (broken) lines indicate major and minor spin channels, respectively.

Fig. 5(a) represents the DFT-optimized crystal structure of YCMO within the *P*2_1_/*n* space group (No. 14). The lattice parameters obtained through PBE-DFT calculations are as follows: *a* = 5.35 Å, *b* = 5.78 Å, *c* = 7.22 Å, and *β* = 89.5°. These values are found to be in good agreement with previous experimental measurements.^15^ The presence of Cu^2+^ in YCMO induces a significant modification in the crystal structure, pushing Mn into a 4^+^ oxidation state, from the more common 3^+^ state observed in YMnO_3_. It also establishes a distinctive superexchange interaction pattern between Mn^4+^ and Cu^2+^ mediated by oxygen (*i.e.*, Mn^4+^–O–Cu^2+^). Structural distortion is expected in these kinds of double perovskites due to the inherent size mismatch between B and B′ ions, and hence lower symmetry structures are preferred. Moreover, the Jahn–Teller active nature of Cu^2+^ (d^9^) ^42^ in YCMO, introduces an additional layer of complexity by enabling pronounced low-symmetry local distortions within the crystal lattice (Fig. 5(b)). These distortions, originating from the unique electronic configuration of Cu^2+^, induce a shift in the crystal structure towards a lower symmetry configuration, ultimately resulting in an orbitally nondegenerate ground state. This phenomenon elucidates the observed stability of the low-symmetry monoclinic structure in YCMO.

#### 3.4.2 Magnetic structure

**Fig. 6:**
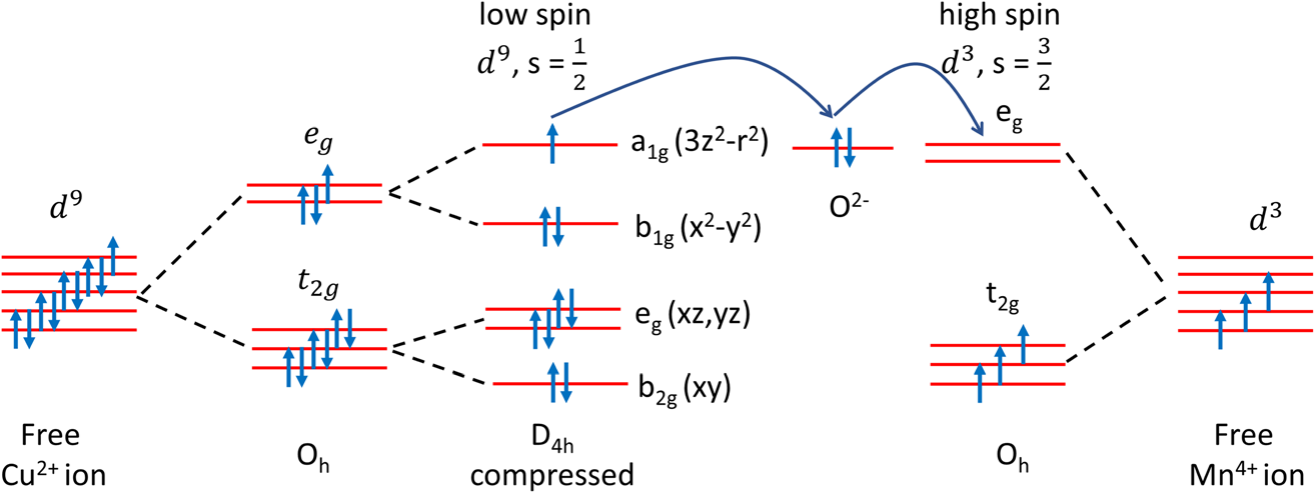
Illustration of Cu^2+^–O–Mn^4+^ superexchange interactions in YCMO.

The stability of the ferromagnetic ground state in YCMO was confirmed by comparing the total energies of various magnetic configurations. According to the Goodenough–Kanamori rules,^43^ the ferromagnetic interactions in YCMO should be attributed to Mn^4+^–O–Cu^2+^ superexchange interactions as illustrated in Fig. 6. The Anderson–Goodenough–Kanamori set of semiempirical rules offers approximate values for superexchange interaction strengths. Following this model, it can be proposed that 180° Mn^4+^–O–Cu^2+^ superexchange interactions exhibit strong ferromagnetism due to the overlap between an empty e_g_ band of Mn^4+^ and a partially filled e_g_ band orbital of Cu^2+^. However, the distortion reduces the Mn^4+^–O–Cu^2+^ bond angle to approximately 145°, leading to expected weak exchange coupling values. This implies that YCMO may demonstrate a low Curie temperature, akin to the isostructural La_2_CuMnO_6_.^44^ The calculated magnetic moments at the Cu and Mn sites are 0.7 *μ*_B_ and 3.1 *μ*_B_, respectively, in the ferromagnetic ground state.

#### 3.4.3 Electronic structure

**Fig. 7:**
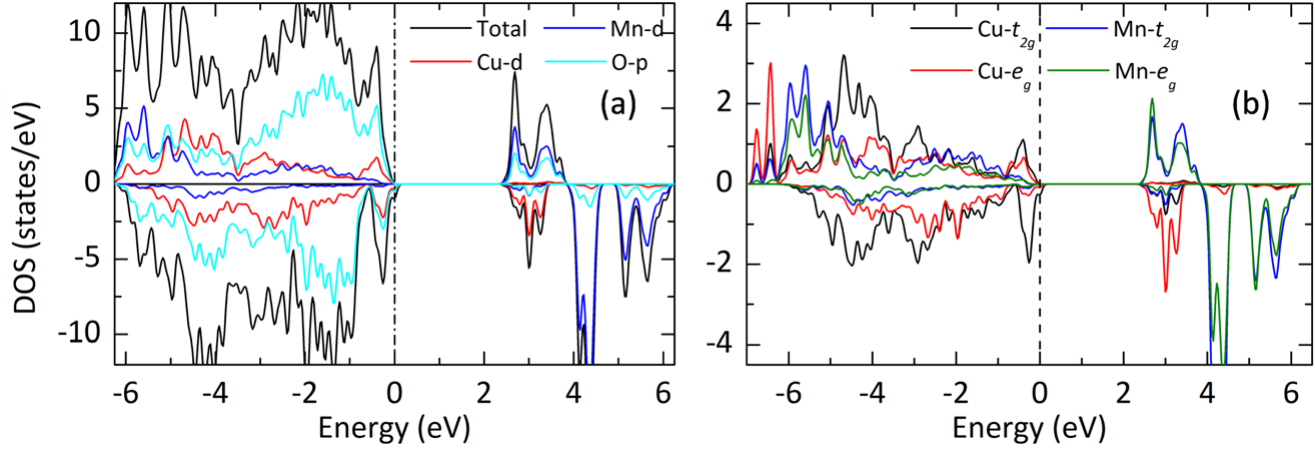
The projected DOS of YCMO: (a) the total and atom-projected DOS and (b) the t_2g_ and e_g_ projected DOS for Mn and Cu.

The spin-polarized electronic band structure (Fig. 5(c)) reveals a band gap of approximately 1.2 eV at the PBE-DFT level of theory. The calculated bandgap is smaller than the measured value due to PBE underestimation. Fig. 7 presents a detailed view of the spin-resolved density of states (DOS) for YCMO calculated with the hybrid HSE functional. The band gap was estimated to be 2.3 eV from the electronic structure. To have a deeper understanding of the electronic structure of YCMO, we have also plotted the atom-projected and Cu/Mn-d orbital projected DOS, as given in Fig. 7(a) and (b), respectively. Under octahedral symmetry, the d orbitals undergo a distinctive splitting into low-energy triply degenerate t_2g_ states and high-energy doubly degenerate e_g_ states. Consequently, the distribution of three d electrons in the Mn^4+^ ion is distributed as t_2g_^3^ and e_g_^0^ configurations. For Cu^2+^, the t_2g_ states are fully occupied, while the e_g_ states have three electrons with a vacant position (as observed by the presence of an empty e_g_ state of Cu^2+^ in the conduction band) in the 3*z*^2^ – *r*^2^ orbital. This unequal distribution in the e_g_ states renders the Cu^2+^ ion Jahn–Teller active, prompting the compression of CuO_6_ octahedra. The resultant distortion induces a further splitting of the Cu-d orbitals, as illustrated in Fig. 6. However, for the sake of simplicity, these orbitals have been represented as t_2g_ and e_g_ in the DOS figure provided in Fig. 7(b).

Additionally, the effective mass of charge carriers is important in semiconductor materials used for photocatalysis as it can impact the rate of charge separation, migration, and overall photocatalytic activity. A lower effective mass generally corresponds to higher carrier mobility, facilitating efficient charge separation and reducing the recombination rate. This is desirable for the effective utilization of photogenerated charges in catalytic reactions. Moreover, the ferromagnetism in YCMO can lead to spin-polarized charge carriers (electrons and holes). The estimated minimum effective mass of electrons at the bottom of the conduction band along Γ–B is 0.8 m_e_, slightly smaller than that of well-known photocatalysts, such as 1 m_e_ for anatase TiO_2_,^45^ and 3–20 m_e_ for rutile TiO_2_.^46,47^ Similarly, the minimum effective mass of holes at the top of the valence band along A–E is 1.03 m_e_, which is smaller than that of other oxide semiconductors, like 16 m_e_ observed in In_2_O_3_.^48^ The low charge carrier masses in YCMO suggest its promising photocatalytic capability.

Spin polarization enables electrons in different spin channels to transition from their respective valence bands to the conduction band, following transition rules when left- and right-handed circularly polarized photons are absorbed. The different alignment of spins of the photogenerated electron–hole pairs can also help in reducing the recombination rate of these charge carriers. This is beneficial for photocatalysis as it increases the number of charge carriers available for redox reactions on the catalyst surface. In addition, when ferromagnetic catalysts are placed in an external magnetic field, electron spin states and external magnetic fields simultaneously affect photocatalysis performance. For example, the photocatalytic performance of α-Fe_2_O_3_/rGO nanocomposites has been shown to improve under the influence of external magnetic fields, with the degradation rate of Rh-B dyes increasing from 59% to 84% as the applied field was raised from 0 kOe to 8 kOe.^49,50^ Similarly, ferromagnetic ZnFe_2_O_4_ exhibited a substantial improvement in photoelectrochemical efficiency, with its photocurrent increasing by 150% when subjected to an external magnetic field of 1 kOe.^49,51^ Moreover, ferromagnetic catalysts can be easily separated from the reaction mixture using an external magnetic field. This makes the catalyst easier to recover and reuse, which is a significant advantage for practical applications. In the next section, we report the photocatalytic properties of YCMO in the degradation of Rh-B and MO dyes. However, the effect of an external magnetic field is not included due to the unavailability of necessary facilities.

### 3.5 Photocatalysis study

Due to the semiconductor properties of the YCMO nanoparticles, they absorb photons from incoming sunlight. Sunlight energy drives electrons from the valence band to the conduction band, leading to the formation of electron–hole pairs. Electron–hole pairs are essential in redox processes. The oxidation of water (H_2_O) molecules by holes in the valence band can result in the formation of reactive oxygen species (ROS), such as superoxide radicals (˙O_2_^−^) and hydroxyl radicals (˙OH). The created ROS are highly reactive and degrade the dye (pollutant) into less harmful compounds.^41^ The photocatalytic dye decomposition is represented by eqn (1) to (5):

1 YCMO → YCMO(e_CB_^−^ + h_VB_^+^)

2 YCMO(h^+^_VB_) + H_2_O → YCMO + H^+^ + ˙OH

3 YCMO(e_CB_^−^) + adsorbed oxygen (O_2_) → YCMO + ˙O_2_^−^

4 Dyes + ˙OH → Degradation → H_2_O + CO_2_

5 Dyes + ˙O_2_^−^ → Degradation → H_2_O + CO_2_

**Fig. 8:**
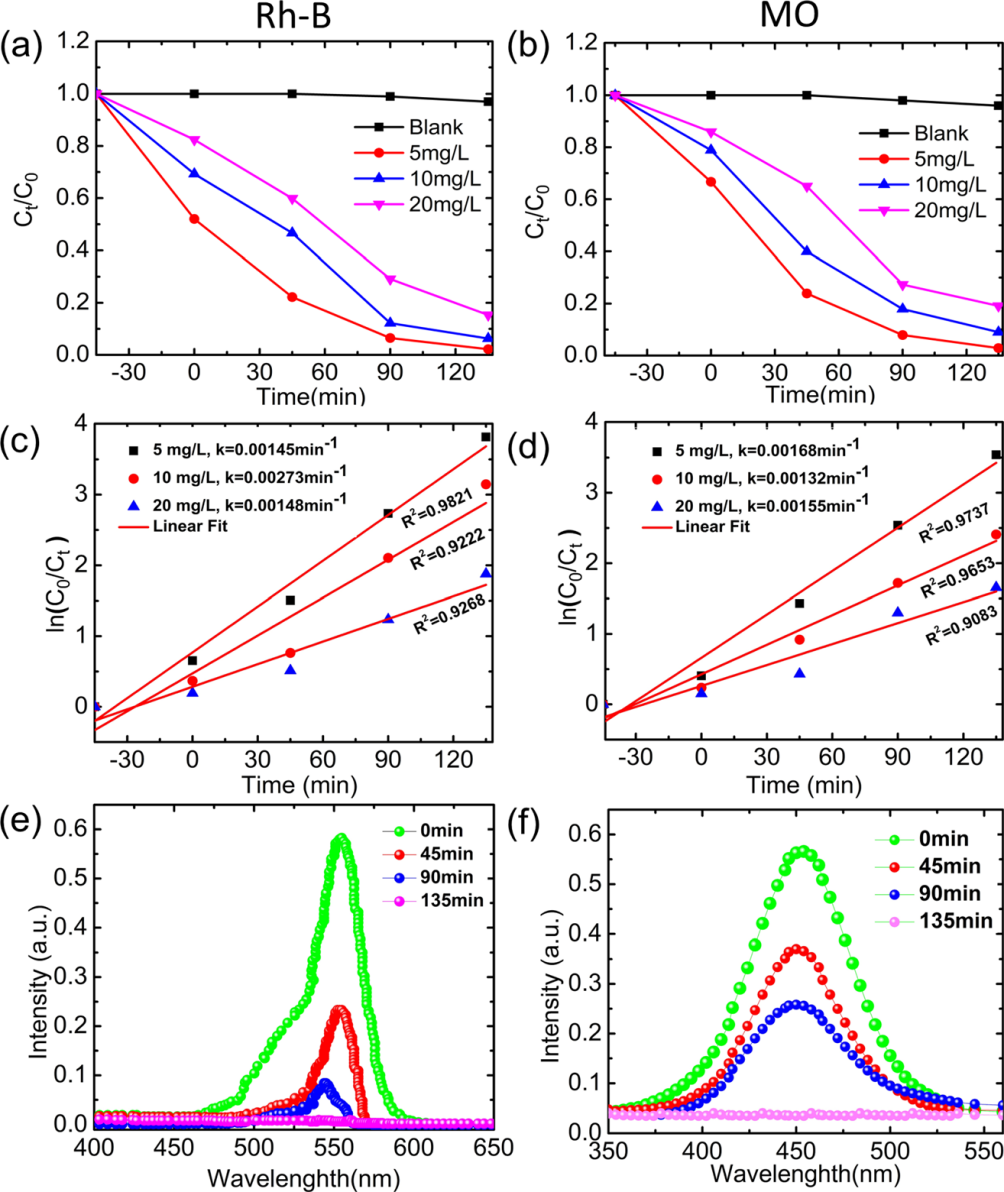
Photocatalytic activity and first-order kinetics calculation of YCMO double perovskite as a function of irradiation time: (a) Rh-B dye degradation, (b) MO dye degradation, (c) first-order kinetics calculation for Rh-B dye, (d) first-order kinetics calculation for MO dye. (e) Time-dependent UV-Vis spectra of Rh-B dye with photocatalyst sample under visible irradiation, and (f) time-dependent UV-Vis spectra of MO dye with photocatalyst sample under visible irradiation.

The degradation of Rh-B and MO dyes was conducted using YCMO nanoparticles as catalysts in the photocatalysis process. Fig. 8(a) and (b) display the results of the photocatalytic degradation tests utilizing YCMO nanoparticles. The test was carried out in direct sunlight to examine the degradation efficiency of the YCMO catalyst using 5 mg L^−1^, 10 mg L^−1^, and 20 mg L^−1^ precursor solutions, resulting in 99%, 93%, and 84% breakdown of Rhodamine B dye and 96%, 90%, and 81% breakdown of MO dye in 135 min, respectively. Interestingly, compared to other traditional photocatalysts, the YCMO compound displayed higher photocatalytic efficiency in the degradation of Rh-B and MO.^52,53^ The plot between ln(*C*_0_/*C*_t_) and irradiation time (t) is represented in Fig. 8(c) and (d), and it is seen that Rh-B and MO dye photodegradation can be effectively modelled using the Langmuir–Hinshelwood first-order kinetic equation, ln(*C*_0_/*C*_t_) = *kt*. This equation properly fits the experimental data, with constants *C*_0_ and *C*_t_ representing the concentration of Rh-B and MO at time zero and time (t), respectively, and *k* as the first-order rate constant. Furthermore, it can be inferred that YCMO at a concentration of 5 mg L^−1^ has the greatest apparent rate constant (*k*), indicating improved photocatalytic activity.

An additional investigation was conducted on the photocatalytic activity of the YCMO double perovskite treated with Rh-B and MO dyes, using different levels of illumination time (as shown in Fig. 8(e) and (f), respectively). The Rh-B and MO dye degradation and periodic UV-Vis spectra showed a progressive decline and a slight blue shift of the characterized band at *λ* = 554 and 464 nm, respectively, suggesting that the synthesized double perovskite completely broke down the dye molecules. Additionally, a decrease in the absorbance values was noted for the relative absorbance band (*λ*_max_), which was detected at 554 and 464 nm, and this further vanished entirely as time extended. This supports the produced double perovskite’s capacity for degrading the Rh-B and MO dyes effectively.

**Fig. 9:**
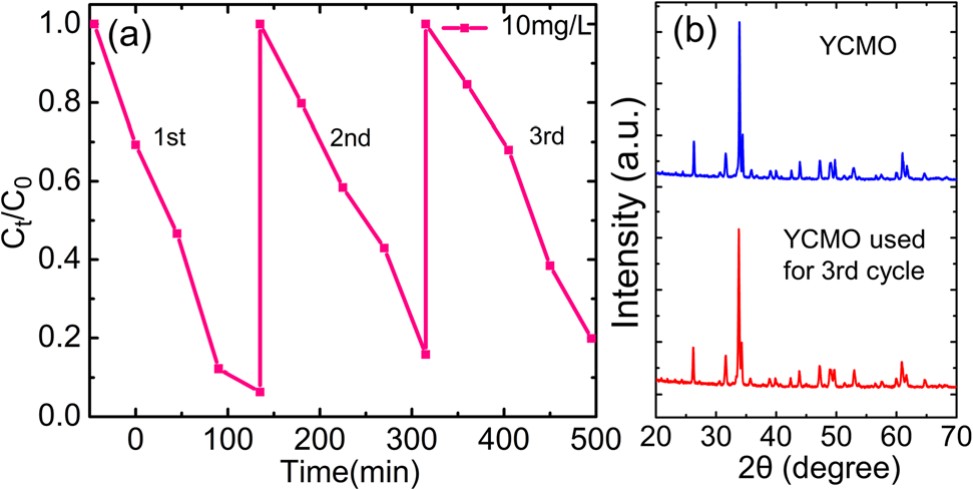
(a) Recycle tests of the photocatalytic degradation of Rh-B solution over the YCMO double perovskite, and (b) comparison of the XRD patterns of the YCMO double perovskite before and after three successive photocatalytic degradation processes.

The practical applications of photocatalysts are largely determined by their stability and reusability. The YCMO double perovskite was put through three successive cycles under the same conditions to measure its stability and reusability. After centrifugation, the mixture was extracted from the photocatalyst, and the remaining Rh-B dye molecules were washed away with ethanol and DI water. After drying, the sample was put back into a new Rh-B dye solution for the following cycle. As seen in Fig. 9(a), the photocatalytic degradation efficiency of the YCMO double perovskite was constant at almost 80%, following the third cycle. The unavoidable loss of sample weight during the collecting procedure is the cause of the small reduction in efficiency. Additionally, there were no significant changes in the phase or structure of the recycled YCMO double perovskite’s XRD patterns before and after the third cycle, as shown in Fig. 9(b), suggesting that the recycled sample maintained its structural stability. The YCMO double perovskite can be employed as a viable and stable photocatalyst for Rh-B degradation.

**Fig. 10:**
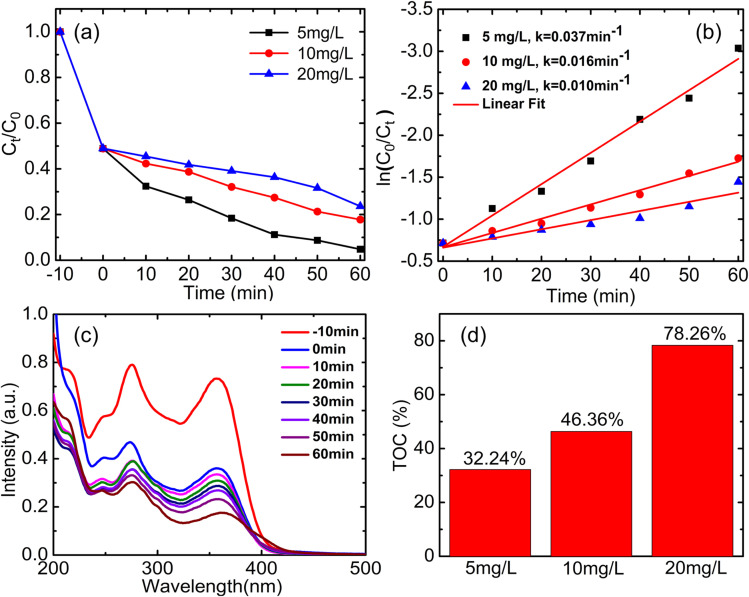
Photocatalytic activity and first-order kinetics calculation of YCMO double perovskite as a function of irradiation time: (a) TC degradation, (b) first-order kinetics calculation for TC, (c) time-dependent UV-Vis spectra of TC with photocatalyst sample under visible irradiation, and (d) TOC removal percentage.

TC antibiotics were degraded using YCMO nanoparticles as the catalysts. Fig. 10(a) displays the results of photocatalytic degradation tests utilizing the YCMO double perovskite. The test was carried out using visible light (500 W halogen lamp) to examine the degradation efficiency of the YCMO catalyst using 5 mg L^−1^, 10 mg L^−1^, and 20 mg L^−1^ precursor solutions, resulting in 95%, 82%, and 76% breakdown of TC antibiotics in 60 min. Interestingly, when compared to other traditional photocatalysts, the YCMO compound displayed higher photocatalytic efficiency in the degradation of TC (shown in Table S2†). The plot of ln(*C*_0_/*C*_t_) and irradiation time (*t*) is presented in Fig. 10(b), using the Langmuir–Hinshelwood first-order kinetic equation. Furthermore, it can be inferred that YCMO at a concentration of 5 mg L^−1^ has the greatest apparent rate constant (*k*), indicating improved photocatalytic activity. The TC degradation using periodic UV-Vis spectra is shown in Fig. 10(c), a progressive decline and a slight blue shift of the characterized band at *λ* = 359 nm, suggests that the synthesized double perovskite completely broke down the TC molecules. Additionally, a decrease in the absorbance values was noted for the relative absorbance band (*λ*_max_), which was detected at 359 nm, and this further vanished entirely over time.^54^ This provides further proof of the capacity of the produced double perovskite to degrade the TC antibiotics effectively. The total amount of organic content present in water after photocatalytic treatment was analysed using TOC as shown in Fig. 10(d). For 5 mg L^−1^ we observe 32.24% organic compound present in post-treatment water, due to the by-product formed from the degradation of TC. Similarly, we observe 46.36 and 78.26% for 10 and 20 mg L^−1^, respectively. The efficacy of the synthesized YCMO photocatalyst was ensured by conducting a comparative study using the commercial P25 (TiO_2_) under similar processing conditions. The detailed comparison of photocatalytic performance for Rh-B, MO dyes, and TC antibiotics under identical experimental conditions is shown in Fig. S1(a and b),† with the corresponding data summarized in Table S1 in the ESI file.†

The performance of YCMO as a photocatalyst demonstrates exceptional efficiency in degrading multiple pollutants, including Rh-B, MO dyes, and TC antibiotics. The synthesized YCMO sample demonstrates competitive performance in dye degradation when compared to traditional photocatalysts such as P25, BiVO_4_, and g-C_3_N_4_ composites, exhibiting comparable or superior activity for MO, Rh-B, and TC degradation (shown in Table S2 in the ESI†). This positions YCMO as a strong alternative to traditional photocatalysts, offering enhanced organic pollutant degradation under similar or less stringent conditions.

## 4. Conclusion

In the present study, the auto-combustion method was used to synthesize YCMO double perovskite. The XRD and Raman investigations revealed the synthesis of YCMO double perovskite in its pure form, exhibiting a monoclinic phase. The electronic structure has also been studied through DFT analysis. Under direct sunlight irradiation, the photocatalytic analysis revealed that the YCMO double perovskite exhibits outstanding photocatalytic activity, resulting in 99%, 96%, and 95% breakdown of Rh-B, MO dyes, and TC antibiotics, respectively. Furthermore, as a catalyst, the YCMO double perovskite demonstrates significant stability throughout the process. The spin-polarized charge carriers in YCMO could be significantly influenced by the application of an external magnetic field, potentially leading to an enhanced photocatalytic response for the degradation of various organic pollutants. This study revealed that the YCMO sample exhibited significantly enhanced photocatalytic activity compared to commercial P25, highlighting its potential for advanced photocatalytic applications. Our study may promote future research into the utilization of the YCMO double perovskite in the field of energy conversion and environmental improvement.

## Conflicts of interest

There are no conflicts to declare.

## Supplementary Material

RA-015-D4RA06357K-s001
