# Supplementary material for: Insights into the electronic, magnetic structure, and photocatalytic activity of Y2CuMnO6 double perovskite
Source: RSC Adv. 2025 Jan 30;15(5):3110–21. doi: 10.1039/d4ra06357k (PMC11781078; doi:10.1039/d4ra06357k)
Supplement: RA-015-D4RA06357K-s001 [file RA-015-D4RA06357K-s001.pdf]

Supplementary Information (SI) for RSC Advances.
This journal is © The Royal Society of Chemistry 2025

Supplementary information

Insights into the Electronic, Magnetic Structure, and Photocatalytic Activity of Y_2CuMnO_6 Double Perovskite

Bhagyashree Munisha,[1] Lokanath Patra,[2] Jyotirmayee Nanda,[3,*] and Sneha Mondal[4]

[1]Centre for Nanoscience and Nanotechnology, ITER, S'O'A Deemed to be University, Khandagiri, Bhubaneswar, 751030, Odisha, India
[2]G. W. Woodruff School of Mechanical Engineering, Georgia Institute of Technology, Atlanta, GA 30332, USA
[3]Department of Physics, ITER, S'O'A Deemed to be University, Khandagiri, Bhubaneswar, 751030, Odisha, India
[4]Department of Physics and Nanotechnology, Faculty of Engineering and Technology, SRM Institute of Science and Technology, Kattankulathur, Chennai, Tamil Nadu, 603203, India
*Corresponding author Email Address: jyotirmayeenanda@soa.ac.in

Figure S1 shows the comparative degradation performance of YCMO and P25 TiO_2 under a halogen lamp. The prepared catalyst YCMO shows faster degradation of organic pollutants in comparison with P25 TiO_2. Table S1 shows the comparison of degradation percentage and rate constant.

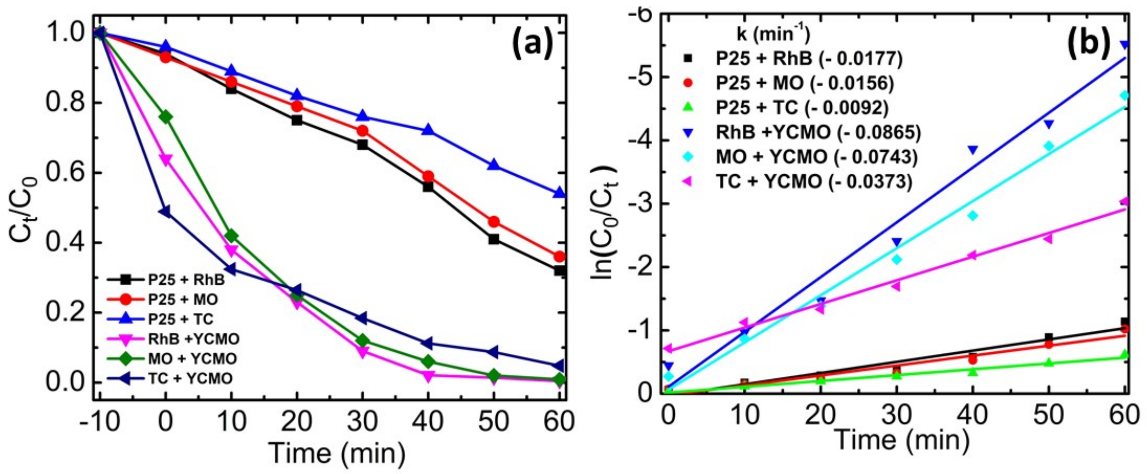

Figure **S1**. Degradation of RhB, MO and TC by P25 TiO_2 and YCMO (a) C_t/C_0 graph and (b) Rate constant.

The lower efficiency of P25 TiO_2 photocatalysts for organic pollutant removal under halogen lamps is primarily due to the spectral characteristics of the light source. Halogen lamps primarily emit in the visible and infrared regions, with only a minimal amount of UV light, which is essential for P25 TiO_2 activation due to its bandgap energy of approximately 3.2 eV. In the absence of sufficient UV radiation, the generation of high-energy charge carriers becomes impossible. Furthermore, the UV light emitted by halogen lamps typically lacks the

intensity and penetration required to activate a substantial portion of the catalyst surface or to generate the reactive oxygen species necessary for the effective degradation of organic pollutants. For optimal performance of P25 TiO_2, light sources with higher UV outputs, such as mercury or UV-LED lamps, are generally preferred.

Table **S1**. Comparative analysis of various pollutant degradation by P25 TiO_2 and YCMO

**Sample**	**Pollutant**	**Degradation Percentage (%)**	**Rate constant (k) (min^{-1})**
P25	RhB	68.4	0.0177
P25	MO	64.7	0.0156
P25	TC	56.2	0.0092
YCMO	RhB	99.8	0.0865
YCMO	MO	99.8	0.0743
YCMO	TC	99.7	0.0373

Table **S2**. Summary of catalyst performance in degradation studies.

**Catalyst Type**	**Synthesis Method**	**Light**	**Concentration (mg/L)**	**Photocatalyst Dosage (mg)**	**Degradation Time (min)**	**Degradation %**	**Ref.**
$Fe\text{-}TiO_2$ /rGO	Sol gel, Nanocomposite	Solar	20	600	120	91% (Rh-B)	[1]
$CuBi_2O_4/ZnBi_2O_4$	Hydrothermal, Hybrid nanostructure	50W LED	10	25	150	60% (Rh-B)	[2]
$g\text{-}C_3N_4/BiVO_4$	Mixed Calcination, Z-Scheme Heterostructure	500 W xenon lamp	--	50	360	85% (Rh-B)	[3]
$Bi_2O_3/g\text{-}C_3N_4$	Hydrothermal, Nanocomposite	Xenon lamp	10	60	150	88.5% (Rh-B)	[4]
$TiO_2/BiVO_4$	Hydrothermal, Nanocomposite	Metal halide lamp with 400	10	10	120	72% (TC)	[5]

		W					
$CeMnO_3$	Citrate sol–gel, Nanoparticles	30 W LED bulb	10	30	90	95% (TC)	[6]
$La_{0.7}Ca_{0.3}MnO_3$	Sol-gel method, Nanoparticles	----	10	5	60	94% (TC)	[7]
Bi_4NbO_8Cl	Molten salt flux, Nanosheets	18W household LED bulb	10	20	60	82% (TC)	[8]
$LaCoO_3/Bi_4Ti_3O_{12}$	Sol-gel, Composites	Xenon lamp	5	25	100	87% (TC)	[9]
Sn–Doped TiO_2	Microwave-assisted sol-gel, Powder	UV visible light	--	5	120	90% (MO)	[10]
ZnO	Co-precipitation method, Nanoparticles	Under sunlight	10	100	300	90% (MO)	[11]
ZnO_G	Forced solvolysis, Nanoparticles	Visible light fluorescent lamp of 160 W/2900 lm LOHUIS	20	12	60	91.07% (MO), 75.43% (RhB), 74.06% (MB)	[12]
Ce-Mo doped $BiVO_4$	Hydrothermal	Visible light (50W halogen lamp; $\lambda > 400$ nm)	10	20	120	85% (MO)	[13]
$BiVO_4$: Mo	Green hydrothermal, Nanoparticles	300 W, visible light illumination using an Xe lamp	10	10	120	86.8% (Rh-B)	[14]
Zn^{2+}: $BiPO_4$	Hydrothermal, Rod-	500 W xenon lamp	20	50	300	81.05% (TC), 91.25%	[15]

	shaped					(RhB)	
CDs/g-$C_3N_4/BiPO_4$	High-temperature calcination, Composite	500 W xenon lamp	10	30	210	75.5% (TC)	[16]
ZrO_2-TiO_2	Sol-gel, Nanocomposites	UV lamp (low-pressure mercury lamp)	30	200	180	75.5% (MO), 78.1% (RhB)	[17]
P-25	---	UV lamp (low-pressure mercury lamp)	20	200	180	90% (MO), 89.8% (RhB)	[17]
TiO_2	Solvothermal route, Nanorods	125 W mercury light	10	50	50	90% (MO), 97% (RhB)	[18]
C@CuNi/TiO_2	Microwave hydrothermal, Composite	500W xenon lamp	10	10	180	94.5% (RhB), 70% (MB), 80% (TC)	[19]
Y_2CuMnO_6	Auto combustion, Double perovskite	Sunlight, Halogen lamp 500W	10	50	135	93% (RhB), 90% (MO), 82% (TC)	This work

References:

[1] A.A. Isari, A. Payan, M. Fattahi, S. Jorfi, B. Kakavandi, Photocatalytic degradation of rhodamine B and real textile wastewater using Fe-doped TiO 2 anchored on reduced graphene oxide (Fe-TiO 2 /rGO): Characterization and feasibility, mechanism and pathway studies, Appl. Surf. Sci. 462 (2018) 549–564. https://doi.org/10.1016/j.apsusc.2018.08.133.

[2] S. et al Srinivasan, D., Karthik, D.R., Arjunan, In situ preparation of Z-scheme CuBi2O4/ZnBi2O4 hybrid nanostructured for enhanced photocatalytic performance,

Emergent Mater. (2024).

[3] N. Tian, H. Huang, Y. He, Y. Guo, T. Zhang, Y. Zhang, Mediator-free direct Z-scheme photocatalytic system: BiVO4/g-C3N4 organic-inorganic hybrid photocatalyst with highly efficient visible-light-induced photocatalytic activity, Dalt. Trans. 44 (2015) 4297–4307. https://doi.org/10.1039/c4dt03905j.

[4] R. Ranjith, S. Vignesh, R. Balachandar, S. Suganthi, V. Raj, S. Ramasundaram, J. Kalyana Sundar, M. Shkir, T.H. Oh, Construction of novel g-C3N4 coupled efficient Bi2O3 nanoparticles for improved Z-scheme photocatalytic removal of environmental wastewater contaminant: Insight mechanism, J. Environ. Manage. 330 (2023) 117134. https://doi.org/10.1016/j.jenvman.2022.117134.

[5] M. Liaqat, S. Kausar, T. Iqbal, S. Afsheen, A. Younas, M. Zubair, A. Syed, A.M. Elgorban, L.S. Wong, Synergistic Photocatalytic Activity of TiO2/BiVO4 Nanocomposites: Optimization, Characterization, and Recyclability for Dye and Antibiotic Degradation, J. Inorg. Organomet. Polym. Mater. 34 (2024) 3115–3126. https://doi.org/10.1007/s10904-024-03102-7.

[6] H.S. Anusha, S. Yadav, T. Tenzin, J.S. Prabagar, K.M. Anilkumar, W. Kitirote, H.P. Shivaraju, Improved CeMnO3 perovskite framework for visible-light-aided degradation of tetracycline hydrochloride antibiotic residue and methylene blue dye, Int. J. Environ. Sci. Technol. 20 (2023) 13519–13534. https://doi.org/10.1007/s13762-022-04742-3.

[7] Z. Li, W. Xie, F. Yao, A. Du, Q. Wang, Z. Guo, H. Gu, Comprehensive electrocatalytic degradation of tetracycline in wastewater by electrospun perovskite manganite nanoparticles supported on carbon nanofibers, Adv. Compos. Hybrid Mater. 5 (2022) 2092–2105. https://doi.org/10.1007/s42114-022-00550-y.

[8] A. Majumdar, A. Pal, Optimized synthesis of Bi4NbO8Cl perovskite nanosheets for enhanced visible light assisted photocatalytic degradation of tetracycline antibiotics, J. Environ. Chem. Eng. 8 (2020) 103645. https://doi.org/10.1016/j.jece.2019.103645.

[9] Z. Zhu, S. Wan, Q. Lu, Q. Zhong, Y. Zhao, Y. Bu, A highly efficient perovskite oxides composite as a functional catalyst for tetracycline degradation, Sep. Purif. Technol. 281 (2022) 119893. https://doi.org/10.1016/j.seppur.2021.119893.

[10] L. Predoană, E.M. Ciobanu, G. Petcu, S. Preda, J. Pandele-Cușu, E.M. Anghel, S.V. Petrescu, D.C. Culiță, A. Băran, V.A. Surdu, B. Ștefan Vasile, A.C. Ianculescu, Photocatalytic Performance of Sn–Doped TiO2 Nanopowders for Photocatalytic

Degradation of Methyl Orange Dye, Catalysts. 13 (2023) 1–23. https://doi.org/10.3390/catal13030534.

[11] M. Asif, M. Shafiq, F. Imtiaz, S. Ahmed, A.A. Alazba, H.N. Hussain, F.N. Butt, S.A. Zainab, M.K. Khan, M. Bilal, Photocatalytic Degradation of Methyl Orange from Aqueous Solution Using ZnO by Response Surface Methodology, Top. Catal. (2024). https://doi.org/10.1007/s11244-024-01969-x.

[12] L. Motelica, O.C. Oprea, B.S. Vasile, A. Ficai, D. Ficai, E. Andronescu, A.M. Holban, Antibacterial Activity of Solvothermal Obtained ZnO Nanoparticles with Different Morphology and Photocatalytic Activity against a Dye Mixture: Methylene Blue, Rhodamine B and Methyl Orange, Int. J. Mol. Sci. 24 (2023). https://doi.org/10.3390/ijms24065677.

[13] S. Sriwichai, S. Sakulsermsuk, S. Duangjam, K. Wetchakun, N. Wetchakun, Synergistic effects of Ce and Mo co-doping for enhancing photocatalytic activity of BiVO4 on methyl orange degradation, Mater. Sci. Semicond. Process. 184 (2024). https://doi.org/10.1016/j.mssp.2024.108791.

[14] C.Y. Tsay, C.Y. Chung, C.Y. Chen, Y.C. Chang, C.J. Chang, J.J. Wu, Enhanced Photocatalytic Performance of Visible-Light-Driven BiVO4 Nanoparticles through W and Mo Substituting, Catalysts. 13 (2023) 475. https://doi.org/10.3390/catal13030475.

[15] A. Chen, N. Wang, R. Lu, H. Zhang, W. Liu, T. Shang, Y. Xu, L. Ruyan, Z. Tian, G. Shijing, D. Jiang, Q. Zhan, Enhanced photodegradation performance of Zn-BiPO4 on RhB and TC under the synergistic effect of oxygen vacancies and built-in electric field, J. Alloys Compd. 1007 (2024) 176418. https://doi.org/10.1016/j.jallcom.2024.176418.

[16] and M.L. Wei Qian, Wangtong Hu , Zhifei Jiang , Yongyi Wu , Zihuan Li , Zenghui Diao, Degradation of Tetracycline Hydrochloride by a Novel CDs/g-C3N4/BiPO4 under Visible-Light Irradiation: Reactivity and Mechanism, Catal. Today. 12 (2022).

[17] V. Ruíz-Santoyo, O.A.G.V. and A.P.-L. , Virginia F. Marañon-Ruiz , Rafael Romero-Toledo, Photocatalytic Degradation of Rhodamine B and Methylene Orange Using TiO2-ZrO2 as Nanocomposite Víctor, Catalysts. 11 (2021) 1–20.

[18] R.B. Rajput, R.B. Kale, Photocatalytic activity of solvothermally synthesized rutile TiO2 nanorods for the removal of water contaminants, Mater. Sci. Eng. B. 294 (2023) 116556. https://doi.org/10.1016/j.mseb.2023.116556.

[19] Y. Yang, H. Shen, C. Ma, D. Li, B. Ma, Y. Lu, Y. Wang, Preparation of C@CuNi/TiO2 with a local surface plasmon resonance for photocatalytic degradation of organic dyes and antibiotics under visible light irradiation, Colloids Surfaces A Physicochem. Eng. Asp. 703 (2024). https://doi.org/10.1016/j.colsurfa.2024.135406.
